# The Impact of Work Stress on Job Satisfaction and Sleep Quality for Couriers in China: The Role of Psychological Capital

**DOI:** 10.3389/fpsyg.2021.730147

**Published:** 2021-12-14

**Authors:** Yujin Xie, Jing Tian, Yang Jiao, Ying Liu, Hong Yu, Lei Shi

**Affiliations:** ^1^Labor Model Health Management Center, Beijing Rehabilitation Hospital, Capital Medical University, Beijing, China; ^2^Department of Health Management, School of Health Management, Harbin Medical University, Harbin, China; ^3^Hospital Office, Beijing Rehabilitation Hospital, Capital Medical University, Beijing, China; ^4^School of Health Management, Southern Medical University, Guangzhou, China

**Keywords:** work stress, job satisfaction, sleep quality, psychological capital, couriers

## Abstract

**Objective:** Work stress is one of the urgent public health problems, which has aroused wide attention. In addition, work stress also has a negative impact on the development of enterprises. This study has three purposes: (1) to understand the current status of working stress among couriers, (2) to examine the association between work stress, job satisfaction and sleep quality of Chinese couriers, and (3) to verify the mediating role of psychological capital.

**Methods:** A cross-sectional survey was conducted among 3000 couriers in Beijing of China from January to December 2019 using cluster stratified random sampling. Descriptive statistical analysis was used to describe demographic characteristics of respondents. Pearson correlation analysis was used to analyze the correlation among work stress, job satisfaction, sleep quality, and psychological capital. The mediating analysis was calculated role of psychological capital.

**Results:** A total of 2831 participants were included in this study. The average score of work stress was 2.49 ± 0.76. Work stress was negatively correlated with job satisfaction (*r* = −0.266, *P* < 0.01), sleep quality (*r* = –0.142, *P* < 0.01), and psychological capital (*r* = −0.268, *P* < 0.01), respectively. The direct effect of work stress on job satisfaction is –0.223, and the total effect of work stress on job satisfaction is –0.267. The a*b*c’ is positive, indicating the mediating role psychological capital has in explaining the relationship between couriers’ work stress and job satisfaction. Moreover, psychological capital plays a mediating role between work stress and sleep quality among couriers.

**Conclusion:** Couriers face certain work stress and should be paid attention to and psychological capital can effectively alleviate the work stress of couriers, so measures can be taken to improve it, promoting healthy development of employees and organizations.

## Introduction

### Work Stress

Work stress is an serious public health problem attracting widespread attention (Margolis et al., 1974). Work stress concerns individuals’ physical reaction caused by an overload of work responsibilities. It occurs when an employee does not have the capability to cope with the current work environment and compromises their physical and mental wellbeing (Seňová and Antosova, 2014). Furthermore, work stress has a detrimental effect on the health of staff with cardiovascular diseases, depression, and cancer (Kivimaki et al., 2006; Melchior et al., 2007; Yang et al., 2019). Work stress negatively impacts organizations, which will ultimately incur losses because of worker dissatisfaction, negativity, high turnover rates, and absenteeism (Webster et al., 2010). Previous studies have focused on the relationship between work stress and burn-out in nursing staff, bank staff, and dentists (Choy and Wong, 2017; Ezenwaji et al., 2019; Giorgi et al., 2019). Other studies have found that work stress impacts employees’ job satisfaction and sleep quality (Lu et al., 2007; Yang et al., 2018).

### Psychological Capital

Luthans et al. (2004) first proposed the concept of psychological capital in 2004. It is an individual’s positive state of psychological development, shown through four principles: self-efficacy, hope, optimism, and resilience. A study found that the psychological capital of employees was positively correlated with job satisfaction (Zhang et al., 2021). A study in China found that work stress and psychological capital affected sleep quality among medical staff in a hospital in Urumqi (Ye et al., 2019). Furthermore, psychological capital is often used in research involving workers from various professions (Chen and Lim, 2012; Kwok et al., 2015), suggesting that couriers are similarly suitable as a topic of study.

### Job Satisfaction

Hoppock (1937) first proposed the concept of job satisfaction and defined it as an “employees’ psychological and physiological satisfaction with the working environment.” He also added that job satisfaction is the subjective reaction of employees to their working environment (Hoppock, 1937). Locke, Spector, and others further defined job satisfaction from different perspectives (Locke, 1969; Spector, 1997; Gigantesco et al., 2003). Studies on job satisfaction have focused on its influencing factors. By summarizing the relevant results, we conclude that job satisfaction is determined by two factors: work environment characteristics, such as work attributes, organizational environment, job type, job security, social status, and promotion prospects, and worker characteristics, such as level of education, gender, age, and personality (Seashore and Taber, 1975). Job satisfaction affects the work performance of the individual members as well as the entire organization. Job characteristics are closely related to job satisfaction and affect employees’ personal life. Stress is a crucial factor that workers in a myriad of occupations experience, and it directly affects job satisfaction (Friganović et al., 2019). This study focuses on the impact of work stress on couriers’ job satisfaction.

### Sleep Quality

Sleep quality is defined in three ways based on sleep indicators. The first–a relatively representative view–uses tools to measure the recorded sleep indicators (Buysse et al., 2006). The second indicator is the sleep quality index, which uses various measuring instruments to assess physiological indicators (Rechtschaffen and Kales, 1968). Third, the quality of sleep is indicated through self-assessment by the sleepers; this indicator is unrelated to the length, frequency, or duration of sleep (Krystal and Edinger, 2008). Buysse et al. assessed sleep quality using the following seven factors: subjective sleep quality, time taken to fall asleep, sleep efficiency, sleep duration, soporific drugs, sleep disorders, and daytime dysfunction. They used the Pittsburgh Sleep Quality (PSQI) index as an indicator to evaluate sleep quality (Buysse et al., 1989). Scholars have recently focused on the physical and psychological effects of sleep quality. Studies have found that lack of sleep leads to poor physical health and exacerbates symptoms of diabetes, coronary heart disease, arrhythmia, anxiety, depression, and negative emotion, thereby affecting one’s mental health (Zhang and Gu, 2001). This study focuses on factors in the work environment influencing the sleep quality of couriers.

### Chinese Couriers

A courier is an employee–responsible for collection, sorting, and delivery–that has direct contact with recipients and senders (Zhao and Liu, 2017). According to the 2019 Statistical Bulletin on the Development of China’s Postal Industry, the express delivery business in China is burgeoning, increasing job opportunities at express delivery enterprises, resulting in a growing need for delivery personnel. Statistically, the number of couriers in China increases by 150,000-200,000 every year (Fang et al., 2017). However, the rapid development of the express delivery industry has precipitated changes in the workforce. Poor courier work ethic, increased resignations, and high staff turnover suggest that, through the period of rapid growth, there should be a focus on enterprise development and increased attention should be paid to courier job satisfaction (Yang et al., 2019). Chinese couriers are generally young and poorly educated. They work long hours on consecutive days in a challenging environment with little to no downtime. The express delivery industry requires little technical knowledge and long working hours while providing no personal growth opportunities. Consequently, it becomes difficult to attract high-performance personnel, resulting in couriers’ relatively low social standing (Yang et al., 2019). Studies have shown work-related stress to be one of the main problems for couriers, with customer complaints being the primary cause of tension (Bian, 2016).

Examining couriers’ work-related stress is beneficial to their occupational health and is socially significant. We used the causal stepwise regression method on the classical mediation test (Baron and Kenny, 1986). The bootstrap test was used to re-examine the role of psychological capital on work stress, job satisfaction, and sleep quality of couriers (Hayes, 2013). To better understand the role of psychological capital on work stress, job satisfaction, and sleep quality among the couriers, we proposed the following hypotheses:

Hypothesis 1: Psychological capital plays a mediating role between work stress and job satisfaction among couriers.
Hypothesis 2: Psychological capital plays a mediating role between work stress and sleep quality among couriers.

## Materials and Methods

### Study Design and Population

This study was conducted jointly by the Beijing federation of trade unions, the Beijing express association, and Beijing Rehabilitation Hospital, Capital Medical University. A cross-sectional survey utilizing an anonymous questionnaire was used to obtain data. Researchers conducted a one-on-one questionnaire survey with each participant with their consent. Cluster random sampling was used to select 10 express delivery companies each in five districts in Beijing. A total of 3,000 couriers from 50 express and logistics companies in Beijing were selected from January 1 to December 31, 2019. A total of 2,831 valid questionnaires were collected, with an effective recovery rate of 94.37%.

The inclusion criteria of participants were: (1) having more than one year’s experience in the express delivery industry, and (2) voluntary and truthful cooperation with the professionals conducting the survey.

### Measurements

The questionnaire involves two main sections: demographic information, such as gender, age, marital status, income, and level of education, and occupational information, including work stress, job satisfaction, sleep quality, and psychological capital.

#### Work Stress Scale

Work stress scale was used to measure couriers’ work stress. It was compiled by Dong (2018), who based it on three studies (Cooper and Marshall, 1976; Ivancevich and Matteson, 1981; Zhang, 2012). The scale is divided into six parts with 24 items: task pressure (five items), job role stress (three items), career development pressure (four items), interpersonal relationship stress (three items), organizational structure and orientation stress (five items), and family-work interaction stress (four items). The scale was scored using a five-point Likert scale, where Strongly disagree, Disagree, Neither agree nor disagree, Agree, and Strongly agree represent 1, 2, 3, 4, and 5, respectively. The higher the score on the scale, the greater the levels of work stress. In this study, the scale’s Cronbach’s alpha was 0.956. The Cronbach’s coefficient of the scale’s six parts were 0.786, 0.891, 0.915, 0.790, 0.904, and 0.9020, respectively.

#### Job Satisfaction

Job satisfaction was assessed using the single measurement item proposed by Wanous et al. (1997), namely “overall, how satisfied are you with your current job.” The measurement also uses the Likert scale from 1, “very dissatisfied” to 5, “very satisfied.” Higher scores indicate higher job satisfaction. This item had adequate reliability and validity in previous research (Dolbier et al., 2005).

#### Sleep Quality

Sleep measurements were assessed using the outcome question of “Over the past month, how do you feel your subjective sleep quality has been?” (Matsumoto et al., 2021). The questionnaire used a rating scale from 1, “very poor” to 5, “very good.” The higher the value, the better the courier’s sleep. The study confirmed that the measurement method has high validity and sensitivity, effectively measuring the courier’s overall perception of sleep quality.

#### Psychological Capital Questionnaires

Psychological capital level was measured using the Psychological Capital Questionnaire (PCQ-24) in this study. It was compiled by Luthans et al. (2008) and translated into Chinese by Chaoping Li in 2008 (Luthans et al., 2007). It has 24 items and four subscales. A score of 1 indicates extreme disagreement, and a score of 6 indicates extreme agreement. The higher the score, the better the level of psychological capital. The Cronbach’s coefficient of the scale was 0.933 in this study.

### Statistical Analysis

In this study, double-entry verification through Epidata 3.1 was used on the questionnaires, and SPSS V25.0 was used for data analysis. Descriptive statistical analysis was used to describe sample information. The Kolmogorov-Smirnov test was used to evaluate the normality of related variables. Control variables were obtained using an independent sample *t*-test and one-way ANOVA to compare the differences between job stress, job satisfaction, psychological capital, and sleep quality scores among participants with different demographic characteristics. Pearson correlation analysis was used to analyze the correlation between work stress, job satisfaction, sleep quality, and psychological capital. The regression and mediating factors were developed using SPSS PROCESS and the macro calculation by Preacher and Hayes (2013). Work stress was the independent variable (X), and psychological capital (M), job satisfaction, and sleep quality were dependent variables (Y). The possibility of multiple collinearities among variables was also considered in this study. *P* < 0.05 was considered statistically significant (two-tailed).

### Ethical Approval

This study was approved by the Ethics Committee of Beijing Rehabilitation Hospital, Capital Medical University. We obtained the verbal consent of each participants involved in the research process.

## Results

### Common Method Biases

Harman’s single factor test was used to identify common method bias. Following the main component analysis, 20 eigenvalues larger than one were recovered. The first factor described the common difference between all elements of the research variables generated by common methods and relationships between research variables. The first factor that explained the variation was 27.89%, which was much lower than the required criterion of 40%. Accordingly, we concluded that there were no serious common method biases in this study.

### Demographics and Characteristics

The demographic characteristics of the participants are shown in Table 1.

**TABLE 1:** Demographic characteristics of the participants (*N* = 2831).

Variables	*N*	Percentage(%)
**Gender**		
Male	2103	74.3
Female	728	25.7
**Age group (years)**		
≤30	1052	37.2
31–40	1150	40.6
≥40	629	22.2
**Marital status**		
Married	2061	72.8
Single/divorced/widowed	770	27.2
**Education level**		
Junior high school or below	742	26.2
High school/technical secondary school	1089	38.5
Junior college or above	1000	35.3
**Monthly income (RMB)**		
≤5000	1307	46.2
5001–8000	1293	45.7
≥8001	231	8.1
**Years of experience**		
≤4	1425	50.3
5–10	1121	39.6
≥11	285	10.1
**Daily working hours**		
≤8	662	23.4
8–12	1895	66.9
≥12	274	9.7

### Correlations Between Study Variables

Table 2 shows the scores of work stress, job satisfaction, sleep quality, and psychological capital. Work stress negatively correlated with job satisfaction (*r* = –0.266, *P* < 0.01), sleep quality (*r* = –0.142, *P* < 0.01), and psychological capital (*r* = –0.268, *P* < 0.01).

**TABLE 2:** Pearson correlation analysis among different variables.

Variables	M ± SD	1	2	3	4
Work stress	2.49 ± 0.76	1			
Job satisfaction	3.76 ± 0.78	–0.266**	1		
Sleep quality	3.25 ± 0.82	–0.141**	0.194**	1	
Psychological capital	4.22 ± 0.66	–0.268**	0.234**	0.148**	1

**P < 0.01.

### Differences of Multivariate Scores Among Participants With Different Demographic Characteristics

There was a significant difference in the job satisfaction scores depending on the couriers’ demographics. The differences among participants with different demographic characteristics in work stress, job satisfaction, sleep quality, and psychological capital are shown in Table 3.

**TABLE 3:** Differences of multivariate scores among participants with different demographic characteristics.

Characteristics	Work stress	Job satisfaction	Sleep quality	Psychological capital
	M ± SD	M ± SD	M ± SD	M ± SD
**Gender**				
Male	2.53 ± 0.77	3.78 ± 0.78	3.26 ± 0.82	4.24 ± 0.67
Female	2.40 ± 0.70	3.70 ± 0.78	3.23 ± 0.82	4.17 ± 0.64
*t*	3.944**	2.353*	0.915	2.550*
**Age group (years)**				
≤30	2.42 ± 0.69	3.73 ± 0.77	3.25 ± 0.81	4.17 ± 0.67
31–40	2.52 ± 0.75	3.76 ± 0.78	3.21 ± 0.84	4.26 ± 0.65
≥40	2.58 ± 0.85	3.81 ± 0.79	3.33 ± 0.80	4.23 ± 0.68
*F*	9.454**	2.103	4.390*	4.454*
**Marital status**				
Married	2.51 ± 0.77	3.77 ± 0.76	3.28 ± 0.81	4.25 ± 0.66
Single/divorced/widowed	2.45 ± 0.70	3.72 ± 0.81	3.19 ± 0.83	4.14 ± 0.67
*t*	1.986*	1.742	2.539*	3.944**
**Education level**				
Junior high school or below	2.56 ± 0.84	3.84 ± 0.82	3.32 ± 0.79	4.25 ± 0.65
High school/Technical secondary school	2.46 ± 0.73	3.75 ± 0.75	3.23 ± 0.82	4.22 ± 0.68
Junior college or above	2.50 ± 0.65	3.62 ± 0.79	3.27 ± 0.89	4.20 ± 0.66
*F*	3.953*	6.700**	2.566	1.402
**Monthly income (RMB)**				
≤5000	2.49 ± 0.76	3.66 ± 0.81	3.23 ± 0.80	4.16 ± 0.66
5001–8000	2.51 ± 0.76	3.82 ± 0.74	3.26 ± 0.82	4.25 ± 0.65
≥8001	2.46 ± 0.71	3.99 ± 0.73	3.33 ± 0.90	4.35 ± 0.69
*F*	0.459	25.762**	1.886	11.235**
**Years of experience**				
≤4	2.51 ± 0.02	3.76 ± 0.02	3.26 ± 0.02	4.17 ± 0.67
5–10	2.46 ± 0.02	3.76 ± 0.02	3.24 ± 0.03	4.29 ± 0.65
≥11	2.54 ± 0.04	3.74 ± 0.05	3.24 ± 0.05	4.22 ± 0.68
*F*	2.037	0.124	0.247	9.932**
**Daily working hours**				
≤8	2.35 ± 0.79	3.90 ± 0.78	3.36 ± 0.79	4.25 ± 0.69
8–12	2.52 ± 0.74	3.73 ± 0.77	3.24 ± 0.82	4.21 ± 0.65
≥12	2.67 ± 0.72	3.56 ± 0.75	3.05 ± 0.86	4.19 ± 0.67
*F*	21.072**	21.842**	13.518**	0.783

*P < 0.05, ** P < 0.01.

### Mediation Regression Models of Study Variables

**FIGURE 1:**
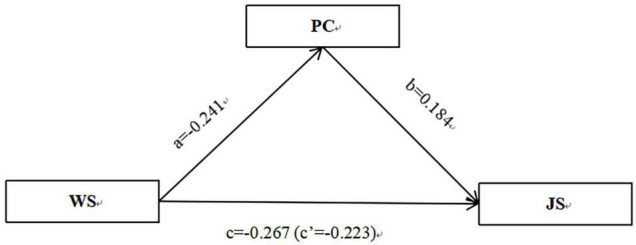
The mediating role of PC in explaining the relation between the WS and JS of couriers. *N* = 2831; controlled for gender, age, educational level, monthly income, and daily working hours. WS, work stress; PC, psychological capital; JS, job satisfaction.

**FIGURE 2:**
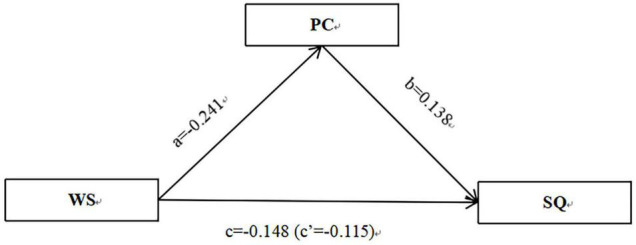
The mediating role of PC in explaining the relation between the WS and SQ of couriers. *N* = 2831; controlled for gender, age, educational level, monthly income, and daily working hours. WS, work stress; PC, psychological capital; SQ, sleep quality.

Let us consider Path 1 (Table 4) as an example for brief overview. The results in Table 4 and Figure 1 can be summarized as follows: the direct effect of work stress on job satisfaction is –0.223, and the total effect of work stress on job satisfaction is –0.267. The a*b*c’ is positive, indicating the mediating role psychological capital has in explaining the relationship between couriers’ work stress and job satisfaction (Figure 1). Path 2 similarly indicates the mediating role of psychological capital (Figure 2).

**TABLE 4:** Results of mediation analyses.

Paths	a	b	c’	a*b	95% CI of a*b	c	SE	R^2^
1. WS → PC → JS	–0.241**	0.184**	–0.223**	–0.044**	(–0.058, –0.032)	–0.267**	0.007	0.136
2. WS → PC → SQ	–0.241**	0.138**	–0.115**	–0.033**	(–0.047, –0.021)	–0.148**	0.008	0.089

N = 2831. Path 1 was controlled for gender, education level, monthly income, and daily working hours. Path 2 was controlled for age, marital status, and daily working hours. WS, work stress; JS, job satisfaction; PC, psychological capital; SQ, sleep quality. *P < 0.05 and **P < 0.01, the indirect effect is significant (*) when the 95% CI does not include 0. SE, bootstrap regression standard error; R^2^, variance accounted for; c’, direct effect; a*b, indirect effect; c, total effect.

## Discussion

A cross-sectional study was conducted to understand work-related stress among couriers, analyze the relationship between work stress, job satisfaction, and sleep quality, and verify the mediating role of psychological capital.

### Current State of Courier Work Stress

This survey found that couriers experience a medium level of work stress, which is lower than the results of other Chinese studies with couriers (Wang et al., 2015). The discrepancy between results may be because of variations between regions and sample sizes. Studies have shown that employees experience feelings of nervousness and unease when they are under pressure at work. This leads to lower work efficiency, lower job satisfaction, resignations, and other phenomena that have a negative impact on personal and organizational development (Kivimaki et al., 2006; Jamal, 2007; Melchior et al., 2007; Webster et al., 2010; Yang et al., 2019). Courier work is tedious, with heavy workloads and low benefits, which only entrenches their work-related stress. This stress is exacerbated by customer distrust and inordinate demands, as well as customer complaints. These external factors influencing couriers’ work stress affect their attitude, job satisfaction, and sleep quality.

### Mediating Role of Psychological Capital

This study shows that psychological capital plays an intermediary role in the relationship between work stress and couriers’ job satisfaction and sleep quality. The results are consistent with other studies (Hayes, 2013). Bolstering psychological capital has a positive effect on work-related stress. It can be regarded as a supplementary mediating effect, indicating that the mediating effect of psychological capital has been established. Couriers are under tremendous pressure. They are overtaxed because of workload and client interaction, causing a physical, mental, and spiritual burn-out. Couriers’ work stress is negatively correlated with their psychological capital. The relationship between psychological capital and work stress is explained according to the resource conservation theory (Hobfoll, 2001). The theory assumes that first, individuals make efforts to acquire, retain, protect, and cultivate valuable resources and minimize resource losses. Second, stress occurs when personal resources are threatened by loss. The work stress will consume the individuals’ favorable resources, ultimately precipitating the negative consequences of the pressure. If the individual has sufficient personal resources to make up for the loss, the adverse effects can be prevented. Studies have confirmed the moderating effect of personal resources. Van Yperen and Snijders (2000) and Pierce and Gardner (2004) found that self-efficacy played a moderating role between job requirements and mental health. Therefore, it can be inferred that psychological capital is a crucial resource. It can balance the negative experiences of courier work, alleviate the resource consumption caused by work-related stress, prevent adverse effects, enhance job satisfaction, and improve sleep quality (Thoits, 1994).

### Implications for Organizational Development

Psychological capital has been shown to have a positive psychological impact on people’s work and home life, and it promotes spontaneous individual growth, facilitating organizational development (Avey et al., 2011). Therefore, improving psychological capital is a necessary intervention to improve couriers’ job satisfaction and sleep quality. Planned psychological training and group counseling can improve couriers’ sense of self-efficacy and self-confidence. These interventions promote self-actualizing behavior, the maintenance of an optimistic attitude, and swift recovery from psychological setbacks. Organizations should particularly focus on the cultivation of couriers’ self-efficacy, confidence, and optimism.

### Limitations

Some limitations in our study should be noted. Our study is cross-sectional. Without a longitudinal survey, the causal relationship between variables cannot be explained. Further longitudinal studies can solve this problem. Moreover, due to the functional characteristics of the express delivery industry, the number of female participants in this study is relatively small. Lastly, the study is limited to couriers only in Beijing. A larger sample needs to be surveyed to verify the generalizability of the study results.

## Conclusion

This study shows that couriers in Beijing experience an medium level of work stress. Measures should be taken to reduce courier work stress to guarantee their job satisfaction and sleep quality. The effect of psychological capital on couriers’ job satisfaction and sleep quality indicates that this variable has practical significance in promoting their health at work and home. It provides a valuable reference for the human resource management of couriers in the future.

## Data Availability Statement

The original contributions presented in the study are included in the article/Supplementary Material, further inquiries can be directed to the corresponding authors.

## Ethics Statement

The studies involving human participants were reviewed and approved by the Ethics Committee of Beijing Rehabilitation Hospital, Capital Medical University. We obtained the consent of each participants involved in the research process. The patients/participants provided their written informed consent to participate in this study.

## Author Contributions

YJ and HY conceived and designed the experiments. YX, YJ, and YL performed the experiments. YX and LS analyzed the data. LS and YL contributed reagents, materials, and analysis tools and provided technical support. YX and JT wrote the manuscript. LS and HY critically revised the manuscript. All authors checked and proofread the final version of the manuscript.

## Conflict of Interest

The authors declare that the research was conducted in the absence of any commercial or financial relationships that could be construed as a potential conflict of interest.

## Publisher’s Note

All claims expressed in this article are solely those of the authors and do not necessarily represent those of their affiliated organizations, or those of the publisher, the editors and the reviewers. Any product that may be evaluated in this article, or claim that may be made by its manufacturer, is not guaranteed or endorsed by the publisher.
