# Supplementary material for: The Impact of Work Stress on Job Satisfaction and Sleep Quality for Couriers in China: The Role of Psychological Capital
Source: Front Psychol. 2021 Dec 14;12:730147. doi: 10.3389/fpsyg.2021.730147 (PMC8712337; doi:10.3389/fpsyg.2021.730147)
Supplement: Supplementary file 1 [file Table_1.DOCX]

**A Questionnaire on Work stress, Job satisfaction, Sleep quality and Psychological Capital of Couriers in Beijing**

Hello!This is a report on the Beijing Courier work stress, job satisfaction, sleep quality and psychological capital. We hope that through this questionnaire to know you for the present work experience and feeling, in order to maintain the healthy development of the industry to provide the reference. We will strictly respect your privacy, the information collected will only be used for academic analysis, not provide to any department or agency divulge. Please you fill out the questionnaire according to actual condition, very thank you for your cooperation! Good luck with your work!

Ⅰ. [Basic](C:/Users/10902/AppData/Local/youdao/dict/Application/8.9.3.0/resultui/html/index.html#/javascript:;) [Information](C:/Users/10902/AppData/Local/youdao/dict/Application/8.9.3.0/resultui/html/index.html#/javascript:;)

Q1. Gender: ①Male ②Female

Q2. Age:_____

Q3. Marital status: ①Married ②Single ③Divorced ④Widowed

Q4. Education level: ①Junior high school or below ②High school or technical ③Secondary school Junior college ④College or above

Q5. Monthly income(RMB): ①≤5000 ②5001-8000 ③≥8001

Q6. Years of experience: ①≤4 ②5-10 ③≥11

Q7. Daily working hours: ①≤8 ②8-12 ③≥12

Ⅱ.Occupational Information

1. The Work Stress Scale

| Questions | Strongly disagree | Disagree | Neutrality | Agree | Strongly agree |
| --- | --- | --- | --- | --- | --- |
| 1.I feel I have a lot of work to do, and I often need to work beyond my capacity. |  |  |  |  |  |
| 2.I feel a lot of responsibility for my job. |  |  |  |  |  |
| 3.I am often asked to finish urgent tasks within a set time. |  |  |  |  |  |
| 4.I always feel overwhelmed by my work. |  |  |  |  |  |
| 5.I often have to work overtime. |  |  |  |  |  |
| 6.I don't feel clear about my job duties. |  |  |  |  |  |
| 7.I am confused by my superior's instructions. |  |  |  |  |  |
| 8.I'm not sure about my goal. |  |  |  |  |  |
| 9.I'm not satisfied with my current position. |  |  |  |  |  |
| 1. My chances for promotion are slim and I worry about future. |  |  |  |  |  |
| 1. I hardly ever get a change of guard. |  |  |  |  |  |
| 1. I couldn't see where my career development and I felt lost. |  |  |  |  |  |
| 1. I often worry that my superiors are not satisfied with my work. |  |  |  |  |  |
| 1. I find it difficult to get along with my colleagues at times. |  |  |  |  |  |
| 1. I often encounter demanding clients and I am afraid of losing them. |  |  |  |  |  |
| 1. I feel the company's performance and compensation system is not reasonable, and I feel under pressure. |  |  |  |  |  |
| 1. I feel that the management of the company is led by the leader. |  |  |  |  |  |
| 1. I felt less support from my boss and felt under pressure. |  |  |  |  |  |
| 1. I felt that none of the ideas and suggestions I put forward to the organization would be accepted. |  |  |  |  |  |
| 1. I think everyone inside the company is very inefficient. |  |  |  |  |  |
| 1. I think sometimes there is a conflict between family life and work. |  |  |  |  |  |
| 1. I feel that my families are not very supportive of my work. |  |  |  |  |  |
| 1. I feel that dealing with family matters will interfere with my work. |  |  |  |  |  |
| 1. I feel the pressure from my family has affected my performance at work. |  |  |  |  |  |

(2)The Job Satisfaction Situation

| Questions | Very dissatisfied | Dissatisfied | Neutrality | Satisfied | Very satisfied |
| --- | --- | --- | --- | --- | --- |
| How satisfied are you with your current job. |  |  |  |  |  |

(3)The Sleep Quality Situation

| Questions | Very poor | Poor | General | Good | Very good |
| --- | --- | --- | --- | --- | --- |
| Status of sleep quality. |  |  |  |  |  |

(4)The Psychological Capital Questionnaire (PCQ-24)

| Questions | strongly disagree | mostly disagree | basically disagree | basically agree | mostly agree | strongly agree |
| --- | --- | --- | --- | --- | --- | --- |
| 1.I feel confident in representing my work area in meetings with management. |  |  |  |  |  |  |
| 2.I feel confident helping to set targets/goals in my work area. |  |  |  |  |  |  |
| 3.I believe I can analyze long-term problems and find solutions. |  |  |  |  |  |  |
| 4.I believe I have contributed to the discussion of the company's strategy. |  |  |  |  |  |  |
| 5.I believe that I have the ability to communicate with people outside the company contact and discuss issues. |  |  |  |  |  |  |
| 6.I believe I can present the information to a group of colleagues. |  |  |  |  |  |  |
| 7.Right now I see myself as being pretty successful at work. |  |  |  |  |  |  |
| 8.If I should find myself in a jam at work, I could think of many ways to get out of it. |  |  |  |  |  |  |
| 9.Right now I am full of energy to complete my work goals. |  |  |  |  |  |  |
| 10.There are many solutions to any problem. |  |  |  |  |  |  |
| 11.I can think of many ways to achieve my present job goal. |  |  |  |  |  |  |
| 12.Right now I am on track to achieve the work goals I set for myself. |  |  |  |  |  |  |
| 13.When I have a setback at work, I have trouble recovering from it, moving on. |  |  |  |  |  |  |
| 14.I usually take stressful things at work in stride. |  |  |  |  |  |  |
| 15.At work, if I have to do it, I can take independent action. |  |  |  |  |  |  |
| 16.At work, I solve problems no matter what. |  |  |  |  |  |  |
| 17.I've been through a lot in the past, so now I'm able to weather the storm at work. |  |  |  |  |  |  |
| 18.In my current job, I feel I can handle many things at once. |  |  |  |  |  |  |
| 19.I always look on the bright side of things regarding my job. |  |  |  |  |  |  |
| 20.If something can go wrong for me work-wise, it will. |  |  |  |  |  |  |
| 21.At work, when it comes to uncertainty, I usually expect the best. |  |  |  |  |  |  |
| 22.I am optimistic about what will happen to my job in the future. |  |  |  |  |  |  |
| 23.In my current job, things have never turned out the way I hoped. |  |  |  |  |  |  |
| 24.At work, I always believe that "behind the darkness is the light, do not be pessimistic". |  |  |  |  |  |  |
